# Broad-Coverage Lipidomics Reveals Temporal Lipid Remodeling and Candidate Serum Biomarkers After Acute Spinal Cord Injury in Rats

**DOI:** 10.3390/biomedicines14092107

**Published:** 2026-09-18

**Authors:** Tengfei Zhao, Hanxu Huang, Chaoran Shi, Feng Zhang, Qionghua Wu, Fangcai Li, Kan Xu

**Affiliations:** 1 Department of Orthopaedic Surgery, The Second Affiliated Hospital, Zhejiang University School of Medicine, Hangzhou 310009, China; 2 Orthopaedic Research Institute of Zhejiang University, Zhejiang University School of Medicine, Hangzhou 310009, China; 3 Key Laboratory of Motor System Disease Research and Precision Therapy of Zhejiang Province, Zhejiang University School of Medicine, Hangzhou 310009, China; 4 Clinical Research Center of Motor System Disease of Zhejiang Province, Zhejiang University School of Medicine, Hangzhou 310009, China

**Keywords:** spinal cord injury, biomarkers, cholesteryl esters, lipidomics, long-chain acylcarnitines, lipid metabolic dysregulation

## Abstract

**Objective:** To systematically characterize temporal lipid metabolic remodeling in serum and spinal cord tissue following spinal cord injury (SCI) and to identify candidate serum lipid biomarkers associated with disease progression. **Methods:** A moderate-to-severe SCI model was established in Sprague-Dawley rats using a modified MASCIS weight-drop contusion protocol. Serum and injured spinal cord tissue were collected at 6 h, 1 d, 3 d, 7 d, and 14 d after injury. Broad-coverage lipidomic profiling was performed using ultra-performance liquid chromatography-tandem mass spectrometry (UPLC-MS/MS). In parallel, transcriptomic profiling of injured spinal cord tissue was conducted, followed by bioinformatic analyses and RT-qPCR validation of selected metabolic genes. **Results:** Broad-coverage lipidomic analysis showed significant and dynamic lipid remodeling in both serum and spinal cord tissue after SCI. The serum lipid profile exhibited a multiphasic pattern, with early suppression, intermediate activation, and late-stage remodeling. Structural phospholipid subclasses showed coordinated temporal changes after injury. Long-chain acylcarnitines (LCACs) increased rapidly in serum during the acute phase and then gradually accumulated in the injured spinal cord tissue. In contrast, cholesteryl esters (CEs) progressively accumulated in spinal cord tissue, while showing a biphasic pattern in serum, with an early decrease followed by a rebound increase. Receiver operating characteristic curve analysis showed that LCACs and CEs had excellent discriminatory performance across multiple post-injury stages. **Conclusions:** SCI is associated with extensive lipid metabolic reprogramming in both serum and spinal cord tissue. LCACs and CEs represent promising candidate serum biomarkers closely associated with disease progression. Multi-omics analyses further suggest that serum lipid remodeling parallels lipid metabolic and inflammatory reprogramming in the injured spinal cord, while the contribution of local pathology to circulating LCAC and CE changes remains to be clarified.

## 1. Introduction

The pathogenesis of traumatic spinal cord injury (SCI) is highly complex and evolves in a stage-dependent manner. Acute SCI is a clinical emergency that commonly results from falls, motor vehicle accidents, sports-related trauma, and violence, and often requires urgent intervention [1]. Depending on the extent of tissue damage, SCI may be classified as complete or incomplete, with clinical outcomes ranging from transient neurological dysfunction to permanent disability or death [2]. Early intervention is therefore critical for limiting tissue damage and improving neurological recovery [3]. However, despite increasing awareness of SCI in military, athletic, and general populations, objective diagnosis, severity stratification, and prognosis prediction remain challenging [1].

Accumulating evidence indicates that biomarkers in serum and cerebrospinal fluid can help delineate injury mechanisms and severity while also informing prognostic assessment after SCI [4,5]. To date, most SCI-related biomarkers are neuron- or astrocyte-associated proteins, such as glial fibrillary acidic protein (GFAP), S100 calcium-binding protein β (S100β), and ubiquitin C-terminal hydrolase L1 (UCH-L1) [6]. However, their clinical translation remains limited by incomplete validation, variable detectability in peripheral blood, disruption of the blood-spinal cord barrier (BSCB), and the relatively specialized requirements for detection. In addition, traditional clinical assessment tools, including the American Spinal Injury Association Impairment Scale, imaging evaluation, and symptom-based examination, are still limited in capturing dynamic molecular and metabolic alterations after SCI [7]. This highlights the need for objective and accessible biomarkers that can reflect disease progression over time.

A growing body of evidence shows that metabolic dysfunction is an important component of secondary injury after SCI [1]. After the primary mechanical injury, the injured spinal cord undergoes marked metabolic remodeling, including mitochondrial dysfunction, oxidative stress, inflammatory activation, and disruption of lipid homeostasis [8,9]. The spinal cord is rich in lipids and depends on tightly regulated lipid metabolism to maintain membrane integrity and cellular function. Therefore, lipid metabolism is especially sensitive to injury-induced disturbance and may reflect ongoing pathological changes within the lesion site [10]. Small-molecule metabolites, especially lipid species, can rapidly respond to tissue injury and may be readily detected in peripheral blood. Comprehensive metabolic profiling during the acute phase of SCI may therefore facilitate the identification of biomarkers associated with injury progression and neurological outcome [11,12]. Ideally, an SCI biomarker should exhibit rapid and measurable changes after injury and should support minimally invasive longitudinal monitoring of disease progression, recovery, and treatment response.

Despite increasing interest in metabolic biomarkers, studies investigating lipid alterations after SCI remain relatively limited [11]. Most previous studies have relied on targeted assays that quantify only a limited number of predefined lipid species or selected metabolic pathways [12,13,14]. Although useful for hypothesis-driven analyses, such approaches are less suited for the unbiased identification of novel lipid alterations and may overlook broader metabolic changes occurring after injury [15]. Consequently, the temporal dynamics of SCI-associated lipid remodeling and the relationship between local pathological events within the injured spinal cord and peripheral metabolic alterations remain poorly understood [14]. Broad-coverage lipidomics provides an opportunity to comprehensively characterize dynamic changes across multiple lipid subclasses in an unbiased manner and may facilitate the identification of serum lipid signatures that can be evaluated in relation to pathological processes within the injured spinal cord [16]. Furthermore, integration of lipidomic and transcriptomic analyses may provide mechanistic insight into how lipid metabolic remodeling interacts with inflammatory signaling and energy metabolism during secondary injury progression [17].

To address these gaps, we employed ultra-performance liquid chromatography-tandem mass spectrometry (UPLC-MS/MS)-based broad-coverage lipidomics to comprehensively characterize temporal lipid remodeling after SCI. Serum and injured spinal cord tissues were collected from adult female rats at multiple time points following moderate SCI to identify candidate lipid biomarkers associated with disease progression and to define their temporal trajectories. In parallel, we performed transcriptomic profiling of injured spinal cord tissue, integrated the transcriptomic data with bioinformatic analyses, and further validated selected metabolic genes by RT-qPCR. Through this multi-omics framework, we aimed not only to identify minimally invasive serum lipid biomarkers, but also to elucidate the molecular basis of lipid metabolic reprogramming during secondary injury progression after SCI.

## 2. Materials and Methods

### 2.1. SCI Model Creation

All animal procedures were conducted in strict accordance with the Guide for the Care and Use of Laboratory Animals and were approved by the institutional ethics committee (ZJCLA-IACUC-20030094). Female Sprague-Dawley (SD) rats weighing approximately 200–220 g were provided by Hangzhou Medical College (Laboratory Animal Use License No. SYXK Zhe 2019-0011). All rats were housed in a controlled environment at approximately 24 °C and 40% humidity under a 12-h light/dark cycle, with three animals per cage. Food and water were provided ad libitum, and bedding was changed daily. All surgical procedures were performed under anesthesia with maintenance of body temperature.

The SCI model was established at the T10 spinal cord level using a previously described Multicenter Animal Spinal Cord Injury Study (MASCIS) weight-drop contusion protocol [18,19,20]. Briefly, 36 SD rats were numbered SD01–SD36 and assigned by an independent investigator using a computer-generated randomization sequence based on a completely randomized design. According to the generated sequence, the animals were allocated to a sham-operated control group and five experimental groups (n = 6 per group). Group assignments were kept sealed to ensure allocation concealment and maintain the integrity of randomization. All rats survived until their predefined euthanasia time points.

Rats in the control group (group 1) were anesthetized, placed in the experimental apparatus, and subjected to a dorsal midline incision with exposure and laminectomy of the vertebral lamina, but no SCI was induced; blood and spinal cord tissue samples were then collected. Rats in the five experimental groups underwent induction of a moderate-to-severe spinal cord contusion under anesthesia using a Multicenter Animal Spinal Cord Injury Study (MASCIS) impactor (Model II, W. M. Keck Center for Collaborative Neuroscience, Rutgers University, Piscataway, NJ, USA). Contusion injury was produced by allowing a 10-g rod to freely fall from a height of 50 mm. Successful model establishment was indicated by immediate bilateral hindlimb twitching after impact and severe locomotor impairment. Locomotor function was assessed in a blinded manner using the Basso, Beattie, and Bresnahan (BBB) scale before sample collection. Serum and spinal cord samples were collected at 6 h, 1 d, 3 d, 7 d, and 14 d after injury, designated as the 6 h, 1 d, 3 d, 7 d, and 14 d groups, respectively. Lipids in serum and spinal cord tissue from 18 rats (3 rats per group) were analyzed by UPLC-MS/MS. RNA sequencing was performed using samples from 15 rats, including 6 control rats and 3 rats from each of the 1 d, 3 d, and 7 d groups. BBB scores in the control, 6 h, 1 d, 3 d, 7 d, and 14 d groups were 21.0 ± 0.0, 0.0 ± 0.0, 0.5 ± 0.2, 1.2 ± 0.3, 3.4 ± 0.8, and 6.2 ± 0.4, respectively.

### 2.2. Preparation of Rat Samples for Lipidomic Analysis

Blood sample collection: Approximately 200 μL of whole blood was collected from the tail vein of each rat using a 20-gauge Precision Glide needle (BD, Franklin Lakes, NJ, USA) and immediately placed on ice. Whole blood samples were then centrifuged at 4 °C and 2500× *g* for 15 min. The supernatant serum was collected, aliquoted into 50-μL fractions, and stored at −80 °C until analysis.

Spinal cord tissue collection: Centered on the lesion epicenter, an approximately 6-mm segment of injured spinal cord tissue was excised along the rostral-caudal axis. After attached blood clots and surrounding tissues had been carefully removed as completely as possible, the specimens were immediately transferred into pre-cooled cryovials. Immediately after collection, the samples were snap-frozen in liquid nitrogen and then stored at −80 °C until analysis.

### 2.3. Broad-Coverage Lipidomics by UPLC-MS/MS

#### 2.3.1. Sample Pretreatment

Tissue samples were removed from −80 °C storage and thawed on ice. Approximately 20 mg of tissue was transferred into a 2-mL centrifuge tube, homogenized with steel beads in a ball mill (30 Hz, 20 s), and briefly centrifuged at 4 °C. The internal standard mixture contained 12:0 lysophosphatidylcholine (12:0 Lyso PC), Cer(d18:1/4:0), and PC(13:0/13:0), each with a purity of >99%. The standards were obtained from Avanti Polar Lipids (Alabaster, AL, USA) and ZZStandard (Shanghai, China). Subsequently, 1 mL of lipid extraction solvent containing internal standards (methyl tert-butyl ether:methanol, 3:1, *v*/*v*) was added, and the mixture was vortexed for 15 min. Then, 200 μL of water was added to promote phase separation, followed by vortexing and centrifugation at 4 °C and 12,000 rpm for 10 min. A 200-μL aliquot of the upper organic phase was collected and evaporated to dryness. The residue was reconstituted in 200 μL of reconstitution solvent (acetonitrile:isopropanol, 1:1, *v*/*v*), centrifuged, and the supernatant was subjected to liquid chromatography-tandem mass spectrometry analysis.

#### 2.3.2. Chromatographic Conditions

Lipid metabolites in serum samples were separated and analyzed using UPLC-MS/MS coupled to an AB Sciex MS/QTRAP 6500+ mass spectrometry system (SCIEX, Framingham, MA, USA). Separation was performed on a Thermo C30 column (2.1 mm × 100.0 mm, 2.6 μm; Thermo Fisher Scientific, Waltham, MA, USA) maintained at 45 °C. The mobile phase was composed of solvent A and solvent B. Solvent A was acetonitrile:water (60:40, *v*/*v*) with 0.04% acetic acid and 5 mmol/L ammonium formate. Solvent B was acetonitrile:isopropanol (10:90, *v*/*v*) with 0.04% acetic acid and 5 mmol/L ammonium formate. The gradient elution program was set as follows: 20% B at 0 min, 50% B at 3 min, 75% B at 9 min, and 90% B at 15 min, followed by re-equilibration to 50% B. The flow rate was 350 μL/min, and the injection volume was 2 μL.

#### 2.3.3. Mass Spectrometric Conditions

The electrospray ionization source temperature was set at 500 °C. The ion spray voltage was 5500 V in positive ion mode and −4500 V in negative ion mode. Ion source gas 1 (GS1) and gas 2 (GS2) were set at 45 psi and 55 psi, respectively, and curtain gas (CUR) was set at 35 psi. Collision-induced dissociation was operated in medium mode. In the triple quadrupole system, each ion pair was scanned under optimized declustering potential and collision energy settings.

#### 2.3.4. Data Quality Control and Multivariate Analysis

##### Sample Quality Control

Quality control (QC) samples were prepared by pooling aliquots of extracts from all samples. During instrumental analysis, one QC sample was typically inserted after every 10 study samples to monitor instrument drift and analytical reproducibility. Overlay of total ion chromatograms from QC samples was used to assess retention time stability and peak intensity consistency. Blank samples were interspersed to evaluate carryover and cross-contamination; no obvious analyte carryover was detected in the blank injections. Correlation analysis among QC samples was also performed. The stability of internal standards in QC samples was used as a process control metric, with a coefficient of variation (CV) of ≤15%. Data quality and batch consistency were further evaluated in combination with the CV distribution in QC samples, the proportion of features with CV < 0.5 exceeding 85% and those with CV < 0.3 exceeding 75%, together with the stability of internal standard responses.

##### Normalization

Multiple reaction monitoring (MRM) chromatographic peaks were integrated and corrected across samples using MultiQuant^TM^ software (version 3.0.2, SCIEX, Framingham, MA, USA), and the resulting peak-area values were used as measures of relative lipid abundance for downstream statistical analysis. Internal standards were included to monitor analytical performance. Because analyte-specific calibration curves were not established for the full lipid panel, the reported values represent relative abundances rather than absolute concentrations. Before multivariate analysis, the data were log2-transformed and mean-centered. OPLS-DA was then used to separate predictive variation from orthogonal variation and evaluate group separation. In the principal component analysis (PCA) and heatmap analyses, unit variance scaling was used to reduce the influence of differences in variable magnitude. This step helped make the variables more comparable; unit variance scaling was used to place lipid features on a comparable scale, reduce the dominance of high-abundance variables, and improve the reliability of the results.

##### Qualitative Identification and Relative Quantification of Lipid Metabolites

To characterize the dynamic changes in lipid metabolism after SCI, qualitative analysis of lipid metabolites was performed on serum and spinal cord samples from the five experimental groups and the control group based on an endogenous metabolite database. Characteristic ion signal intensities were acquired by the detector in counts per second, and chromatographic peaks were integrated and corrected using MultiQuant software. Relative abundances of lipid metabolites in serum and spinal cord tissue were calculated from the peak areas of the corresponding chromatographic signals in the mass spectrometric data and were compared across the six groups. Raw data were processed using Analyst 1.6.3 software. Key metabolites associated with enzymes and upstream genes were further screened using the Human Metabolome Database (HMDB).

##### Criteria for Screening Differentially Abundant Lipid Metabolites

Differential analysis was performed by pairwise comparison of each post-injury group (6 h, 1 d, 3 d, 7 d, and 14 d) against the control group. For each comparison, an orthogonal partial least squares discriminant analysis (OPLS-DA) model was built. Variable importance in projection (VIP) scores were then used to estimate how much each lipid contributed to the separation between groups. Higher VIP scores indicated a larger contribution. In parallel, two-sided *t*-tests were performed to calculate *p*-values for each lipid species, followed by Benjamini–Hochberg correction to control the false discovery rate (FDR). Differentially abundant lipids were identified by integrating fold change (FC), VIP, and FDR using the following thresholds: FC > 1.2 or FC < 0.83, VIP > 1.0, and FDR < 0.05. These combined criteria were selected with reference to commonly used screening strategies in lipidomics and metabolomics studies.

##### Lipid Subclass Analysis

Broad-coverage lipidomic profiling of serum lipids was performed in MRM mode, and lipid subclasses were assigned and summarized according to primary and secondary classifications (Class I/Class II). At the subclass level, lipids were broadly categorized into fatty acyls, glycerophospholipids, sphingolipids, glycerolipids, and sterol lipids. For each lipid species, the corrected MRM peak area was used as a measure of relative abundance for between-group statistical analysis.

### 2.4. RNA Sequencing

#### 2.4.1. Tissue Collection and Total RNA Extraction

Experimental SCI was induced using the same MASCIS impactor protocol described above. At 1 d, 3 d, and 7 d after injury, an approximately 6-mm segment of spinal cord tissue centered on the lesion epicenter was collected, immediately snap-frozen in liquid nitrogen, and stored at low temperature until further processing. Total RNA was extracted from the spinal cord tissue samples using TRIzol reagent (Cat. No. 15596026, Invitrogen, Carlsbad, CA, USA) according to the manufacturer’s instructions.

#### 2.4.2. Library Construction and Sequencing

After RNA samples passed quality assessment, mRNA was isolated and reverse-transcribed into complementary DNA (cDNA). Library preparation then included end repair, A-tailing, adapter ligation, purification, PCR amplification, denaturation, and circularization. The completed libraries were first quantified with a Qubit 4.0 Fluorometer (Thermo Fisher Scientific, Waltham, MA, USA) and insert size was checked using the Agilent 2100 Bioanalyzer (Agilent Technologies, Inc., Santa Clara, CA, USA) to confirm library quality. Qualified indexed libraries were then clustered and sequenced on the Illumina NovaSeq platform, generating 150-bp paired-end reads.

#### 2.4.3. Transcriptomic Data Analysis

Raw sequencing data were subjected to quality control to obtain clean reads, which were then aligned to the rat reference genome. Sequence alignment was performed using HISAT2 (version 2.2.1), and gene-level read counts were generated using featureCounts (version 2.0.3, Subread package). Differential expression analysis was performed using DESeq2 (version 1.38.3), followed by Benjamini–Hochberg correction for multiple testing. Differentially expressed genes (DEGs) were identified using the thresholds of |log2 fold change| ≥ 1 and FDR < 0.05. To assess global expression patterns and within-group reproducibility, PCA and sample correlation analysis were performed on the normalized expression matrix.

To characterize the dynamic evolution of transcriptional programs after SCI, transcriptomic data obtained at 1 d, 3 d, and 7 d after injury were analyzed in a temporal framework. Specifically, the 1 d, 3 d, and 7 d post-injury groups were each compared separately with the control group for differential expression analysis using DESeq2. DEGs identified from these three comparisons, including both upregulated and downregulated genes, were then used to construct a Venn diagram to visualize shared and time point-specific DEGs across the post-injury stages. Gene Ontology (GO) enrichment analysis was then performed on the DEGs identified at each time point to characterize their functional distribution, particularly with respect to lipid metabolism-related and other relevant biological processes. In addition, gene set enrichment analysis (GSEA) was applied to ranked gene lists from each time point to assess pathway-level alterations across the transcriptome and to identify biological programs that remained consistently activated or suppressed after injury. GO enrichment analysis and GSEA were both implemented using clusterProfiler (version 4.6.0, R package), and significance was determined on the basis of adjusted FDR values.

### 2.5. RT-qPCR

Selected genes were validated by reverse transcription-quantitative polymerase chain reaction (RT-qPCR). Complementary DNA generated by reverse transcription was used as the template for amplification with AG SYBR Green qPCR Mix on an Applied Biosystems 7500 Real-Time PCR System (Applied Biosystems, Foster City, CA, USA). Each 10-μL reaction contained 5 μL of 2× AG SYBR Green qPCR Mix, 0.4 μL each of forward and reverse primers (10 μmol/L), 1 μL of diluted cDNA, and nuclease-free water to the final volume. The amplification program consisted of an initial denaturation at 95 °C for 30 s, followed by 40 cycles of 95 °C for 5 s and 60 °C for 30 s. A melting curve analysis from 65 °C to 95 °C was performed at the end of amplification to confirm a single specific product.

Primers were designed using NCBI Primer-BLAST (which uses Primer3 version 2.5.0; https://www.ncbi.nlm.nih.gov/tools/primer-blast/, accessed on 7 September 2026). Amplicon lengths ranged from 80 to 200 bp, and amplification efficiencies ranged from 90% to 110%. Candidate genes for RT-qPCR were selected from the RNA-seq data based on significant differential expression at two or more post-injury time points relative to the control group (|log2 fold change| ≥ 1 and FDR < 0.05), functional relevance to major metabolic pathways, and representation of distinct temporal expression patterns. The RT-qPCR panel included 7-dehydrocholesterol reductase (*Dhcr7*), 3-hydroxy-3-methylglutaryl-CoA reductase (*Hmgcr*), 3-hydroxy-3-methylglutaryl-CoA synthase 1 (*Hmgcs1*), 3-hydroxy-3-methylglutaryl-CoA synthase 2 (*Hmgcs2*), isopentenyl-diphosphate delta isomerase 1 (*Idi1*), nuclear factor, erythroid 2 (*Nfe2*), and enoyl-CoA hydratase and 3-hydroxyacyl-CoA dehydrogenase (*Ehhadh*). The primer sequences are listed in Table 1. Glyceraldehyde-3-phosphate dehydrogenase (Gapdh), a commonly used housekeeping gene, was used as the internal reference gene.

Each group included at least three biological replicates, and each gene was analyzed with three technical replicates. Cycle threshold (Ct) values were exported using a unified threshold setting, and relative gene expression levels were calculated using the 2^−ΔΔCt^ method and normalized to the control group.

### 2.6. Statistical Analysis

Statistical analyses and data visualization were performed using R software (version 3.5.3). Continuous variables were tested for normality using the Shapiro–Wilk test, and normally distributed data were presented as the mean ± standard deviation. Comparisons among multiple groups were performed using one-way analysis of variance, followed by Tukey’s post hoc test for multiple comparisons. For differential lipid analysis, each post-injury time-point group was compared separately with the control group to calculate FC values and corresponding *p*-values, with multiple testing controlled by FDR. Volcano plots displayed log2(FC) on the *x*-axis and −log10(*p*-value) on the *y*-axis. Differential status was assigned using the FC, VIP, and FDR thresholds defined above.

Diagnostic performance was evaluated using receiver operating characteristic (ROC) curve analysis, and the area under the curve (AUC) was calculated. The optimal cutoff value was determined using the Youden index, and the corresponding sensitivity and specificity were reported. Where appropriate, DeLong’s test was used to compare AUC values, followed by FDR correction. All tests were two-sided, with α = 0.05.

## 3. Results

### 3.1. Temporal Distribution of Differentially Expressed Serum Lipids and Kyoto Encyclopedia of Genes and Genomes (KEGG) Pathway Enrichment Analysis After SCI

**Figure 1:**
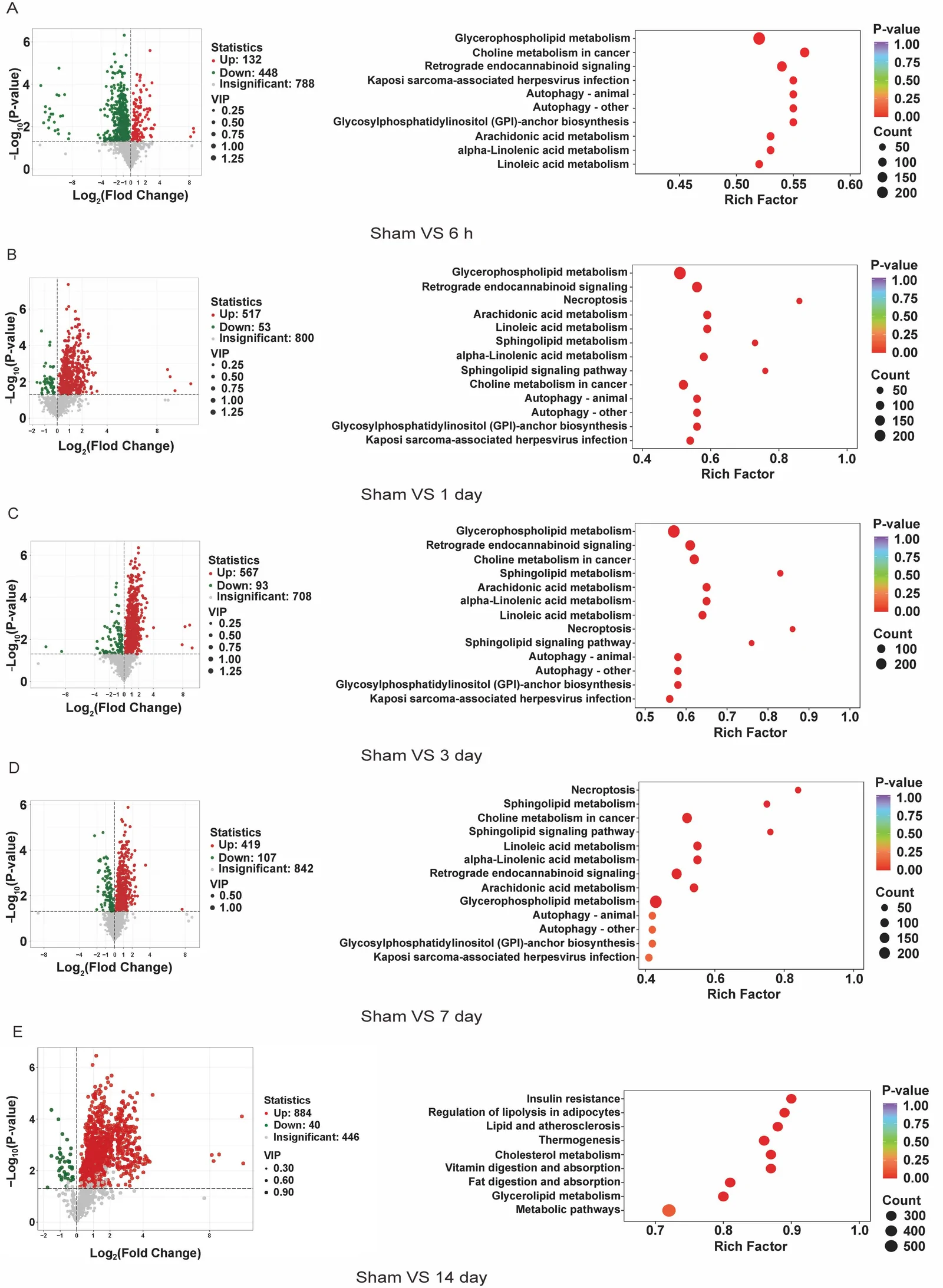
Distribution of differentially abundant lipids in serum and KEGG pathway enrichment analysis at different time points after SCI. (**A**–**E**). Differential lipid profiles were compared between the control group and the 6 h, 1 d, 3 d, 7 d, and 14 d groups. In the left panels, volcano plots display differentially abundant lipids, with log2(FC) shown on the *x*-axis and −log10(*p*-value) on the *y*-axis. Upregulated lipids are shown in red, downregulated lipids in green, and lipids without significant changes in gray; the dot size represents the variable importance in projection (VIP) value. In the right panels, KEGG pathway enrichment of the differentially abundant lipids is presented as bubble plots. The *x*-axis represents enrichment strength, and the *y*-axis lists the enriched pathways. Bubble size corresponds to the number of differentially abundant lipids mapped to each pathway, whereas bubble color reflects statistical significance.

Based on the predefined criteria for screening differentially abundant lipid metabolites, a total of 1372 lipid species were detected and relatively quantified, encompassing five major lipid categories and 19 subclasses, revealing extensive temporal remodeling of the serum lipidome after SCI (Figure 1). The 6 h post-injury group was characterized predominantly by lipid downregulation; compared with the control group, 448 of 580 differentially abundant lipids were decreased. From 1 d afterinjury onward, this pattern reversed, with 517 upregulated and 53 downregulatedlipids. At 3 d, 660 lipids were differentially abundant (567 upregulated and 93downregulated), and upregulation remained predominant at 7 d (419 upregulated and107 downregulated). At 14 d, the original volcano plot identified 884 upregulated and 40downregulated lipids, corresponding to 924 differentially abundant lipids; 446 lipidswere classified as insignificant. Thus, the displayed differential lipid profiles showedpredominant downregulation at 6 h followed by predominant upregulation from 1 dthrough 14 d.

KEGG pathway enrichment analysis of differentially abundant lipids further supported the temporal remodeling of the serum lipidome. In the 6 h group, altered lipids were predominantly enriched in pathways related to membrane lipid remodeling and lipid signaling, including arachidonic acid-derived lipid mediator metabolism, as well as pathways associated with autophagosome biogenesis. From 1 d to 7 d after injury, these membrane lipid- and lipid mediator-related pathways remained prominently enriched, whereas necroptosis-related pathways were consistently enriched at multiple time points. By 14 d after injury, pathway enrichment shifted toward systemic metabolic processes and energy homeostasis, suggesting that late-stage changes were more strongly associated with global metabolic reprogramming.

### 3.2. Temporal Remodeling of Major Lipid Subclasses After SCI

**Figure 2:**
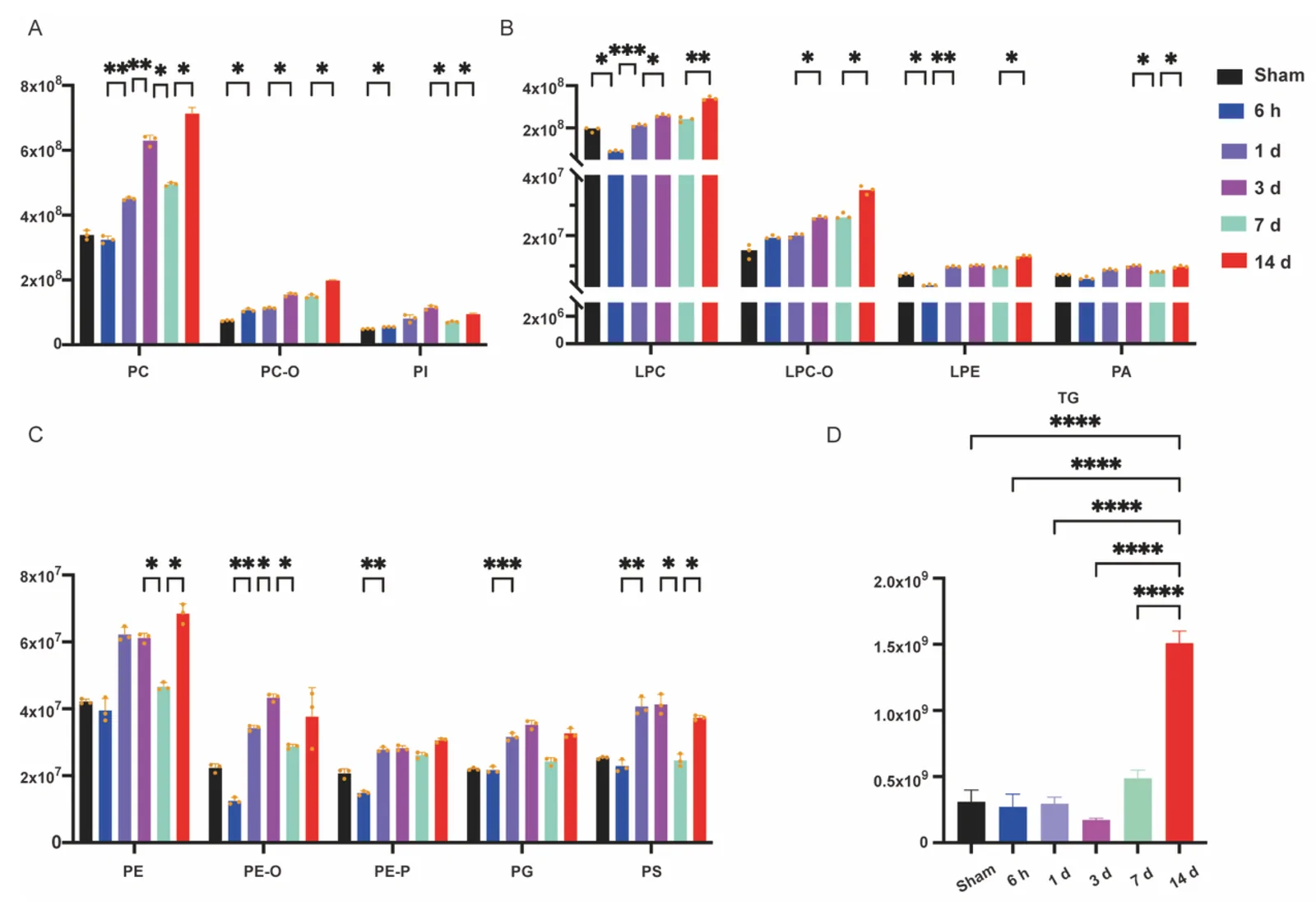
Temporal changes in representative serum lipid subclasses after SCI. (**A**–**C**). Relative abundance of major glycerophospholipid-related subclasses in the 6 h, 1 d, 3 d, 7 d, and 14 d groups, each compared with the control group. (**D**). Time-dependent changes in the relative abundance of triglycerides. PC, phosphatidylcholine; PC-O, ether phosphatidylcholine; PE, phosphatidylethanolamine; PE-O, ether phosphatidylethanolamine; PE-P, plasmalogen phosphatidylethanolamine; PI, phosphatidylinositol; PG, phosphatidylglycerol; LPC, lysophosphatidylcholine; LPC-O, ether lysophosphatidylcholine; LPE, lysophosphatidylethanolamine; PA, phosphatidic acid; PS, phosphatidylserine; TG, triglyceride; SCI, spinal cord injury. * *p* < 0.05, ** *p* < 0.01, *** *p* < 0.001; **** *p* < 0.0001. Each orange dot represents an individual rat.

At the lipid subclass level, SCI induced extensive remodeling of serum glycerophospholipids (Figure 2). Structural phospholipid subclasses exhibited coordinated temporal changes after injury. Levels of phosphatidylcholine (PC) and phosphatidylethanolamine (PE) increased significantly as early as 1–3 d after injury; although they showed a partial decline at 7 d, they remained higher than those in the control group and rose again at 14 d (Figure 2A,C). Phosphatidic acid (PA) and phosphatidylserine (PS) exhibited increased levels at 1–3 d, returned to values close to those of the control group at 7 d, and were elevated again at 14 d (Figure 2B,C). A similar temporal profile was observed for ether-linked phospholipid subclasses, including ether phosphatidylcholine (PC-O), ether phosphatidylethanolamine (PE-O), plasmalogen phosphatidylethanolamine (PE-P), phosphatidylinositol (PI), and phosphatidylglycerol (PG). Levels of these lipid subclasses increased during the early phase after injury and remained elevated through 7 d and 14 d (Figure 2A,C). Progressive alterations were also observed in lysophospholipid subclasses after SCI. This was particularly evident for lysophosphatidylcholine (LPC), which increased steadily throughout the observation period, whereas ether lysophosphatidylcholine (LPC-O) and lysophosphatidylethanolamine (LPE) exhibited more pronounced elevations at later time points (Figure 2B). It is worth noting that triglyceride (TG) shows a different pattern from the above phospholipid subclasses. Within 7 days after injury, the TG level remained relatively stable, but increased significantly at 14 d, suggesting that progressive neutral lipid accumulation may occur in the subacute and late phases of SCI (Figure 2D).

### 3.3. Temporal Dynamics of Cholesteryl Esters and Long-Chain Acylcarnitines After SCI

**Figure 3:**
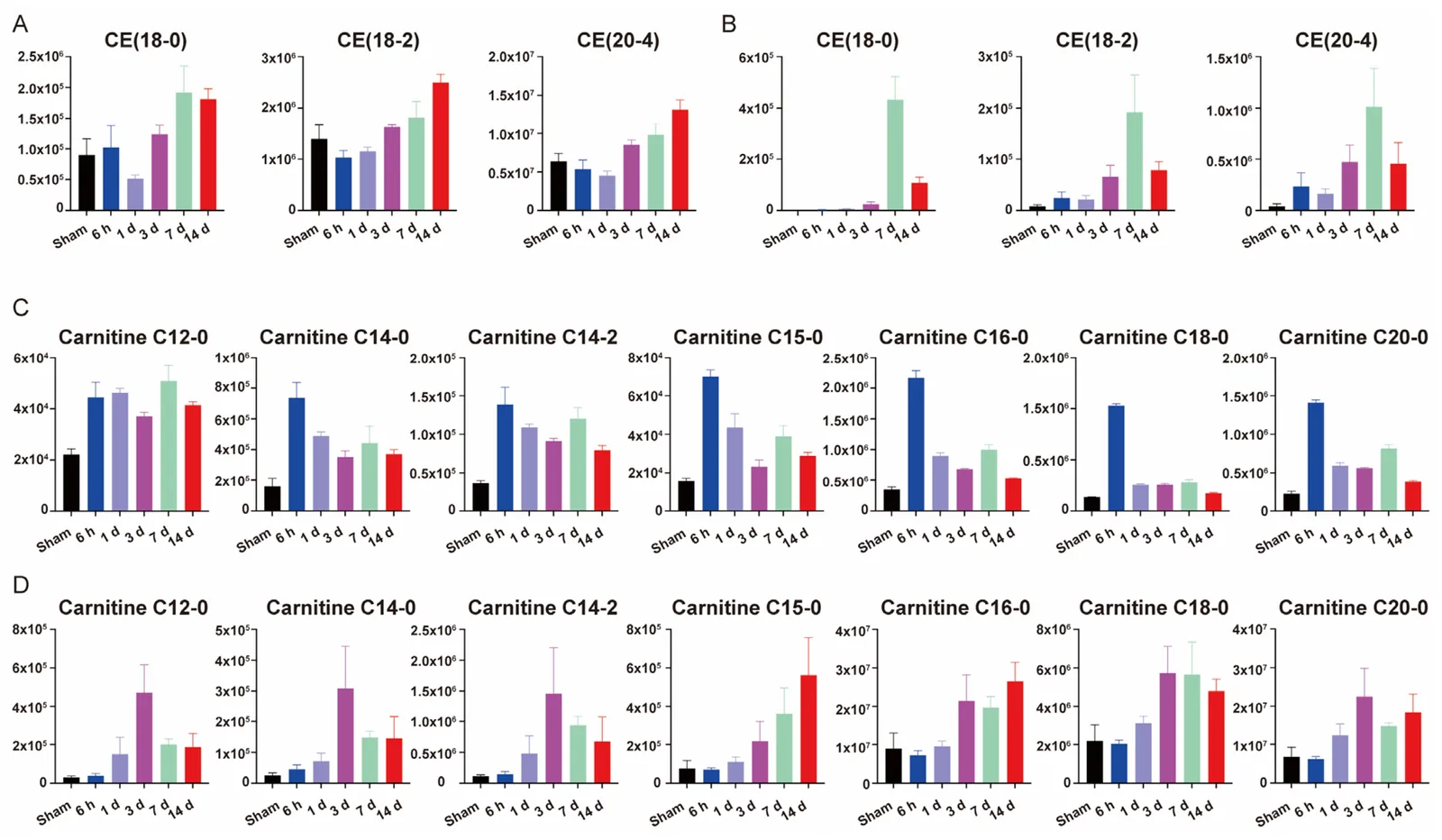
Relative abundance of selected lipid species at 6 h, 1 d, 3 d, 7 d, and 14 d after SCI in rats, as determined by broad-coverage lipidomics. (**A**). In serum, cholesteryl esters (CEs; CE18:0, CE18:2, and CE20:4) showed a biphasic trend, with an early decrease followed by a later increase. (**B**). In spinal cord tissue, CEs increased from 6 h after injury, reached a peak at 7 d, and then declined at 14 d, with the highest relative abundance observed at 7 d. (**C**). Serum long-chain acylcarnitines (LCACs; carnitine C12:0, C14:0, C14:1, C15:0, C16:0, C18:0, and C20:0) were markedly elevated at 6 h after injury and then gradually decreased over time. (**D**). In the spinal cord, LCAC accumulation was delayed, with significant elevations predominantly observed starting at 3 d. Values represent corrected MRM peak areas and are presented as relative abundances rather than absolute concentrations.

The analysis of selected lipid molecules further revealed the distinct temporal patterns of lipid dysregulation after SCI (Figure 3). In serum, several cholesteryl esters (CE18:0, CE18:2 and CE20:4) decreased at first and then increased, with a clear rise from 3 d after injury onward (Figure 3A). In contrast, in spinal cord tissue, the average levels of cholesteryl esters (CE18:0, CE18:2 and CE20:4) were significantly higher than those in controls at 7 d after injury (Figure 3B). These results show that CE changes over time differ between the circulation and the injured spinal cord after SCI. Serum long-chain acylcarnitines (LCACs), including Carnitine C12:0, C14:0, C14:1, C15:0, C16:0, C18:0 and C20:0, increased significantly during the acute phase after SCI, and then gradually decreased over time (Figure 3C). In contrast, LCACs progressively accumulated in the spinal cord and increased significantly from 3 d after injury onward (Figure 3D).

### 3.4. Exploratory Diagnostic Performance of Candidate Serum Lipid Biomarkers After SCI

**Figure 4:**
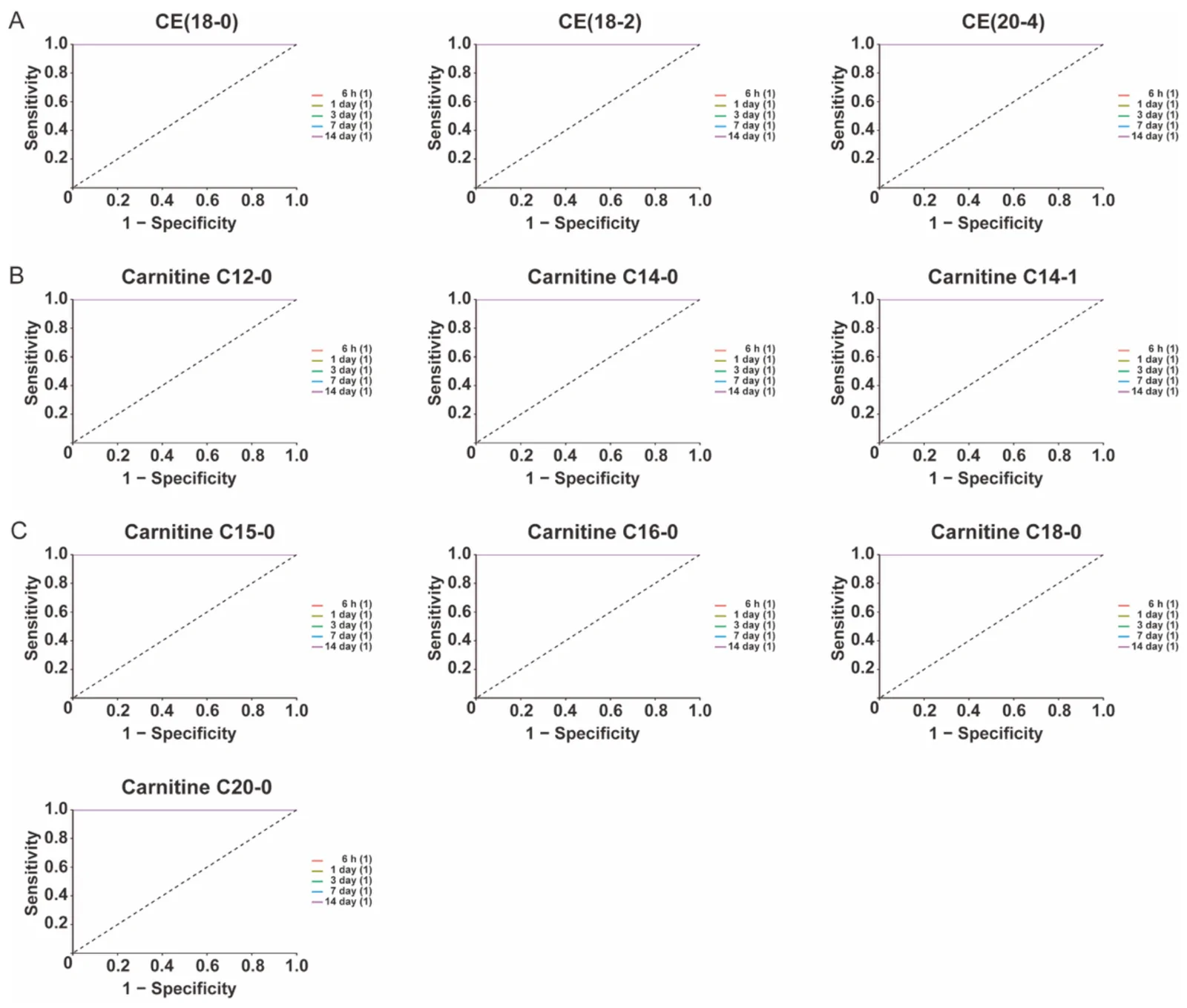
ROC analysis of candidate serum lipid biomarkers after SCI. (**A**) Receiver operating characteristic (ROC) curves for serum cholesteryl esters, including CE18:0, CE18:2, and CE20:4. (**B**,**C**). ROC curves for serum long-chain acylcarnitines (LCACs), presented according to increasing carbon chain length: carnitine C12:0, C14:0, C14:1, C15:0, C16:0, C18:0, and C20:0. In each panel, ROC curves were generated by comparing the 6 h, 1 d, 3 d, 7 d, and 14 d groups separately with the sham group.

Receiver operating characteristic (ROC) curve analysis was used to assess the within-dataset discriminatory performance of candidate lipid biomarkers after SCI (Figure 4). Overall, these candidate serum lipid biomarkers showed strong discrimination within the present dataset throughout the observation period. Serum cholesteryl esters (CE18:0, CE18:2 and CE20:4) yielded an AUC of 1.00 at each examined post-injury time point when compared with the control group (Figure 4A). Similarly, serum long-chain acylcarnitines (LCACs), including Carnitine C12:0, C14:0, C14:1, C15:0, C16:0, C18:0 and C20:0, also showed outstanding diagnostic performance throughout the post-injury observation period. All AUC values were 1.00 (Figure 4). Notably, robust diagnostic performance of LCACs was already evident at 6 h after injury, suggesting that changes in circulating LCACs occur rapidly after SCI and may provide an early metabolic signature of injury. Importantly, the high diagnostic accuracy of both CE and LCAC subclasses was maintained from 6 h to 14 d after injury, warranting further evaluation of their potential value for early detection and longitudinal monitoring in independent cohorts.

Collectively, these findings identify CEs and LCACs as promising serum biomarkers for SCI, with high sensitivity and specificity across multiple post-injury stages.

### 3.5. Transcriptomic and RT-qPCR Analyses Reveal Sustained Lipid Metabolic Reprogramming and a Stage-Dependent Metabolic Shift After SCI

**Figure 5:**
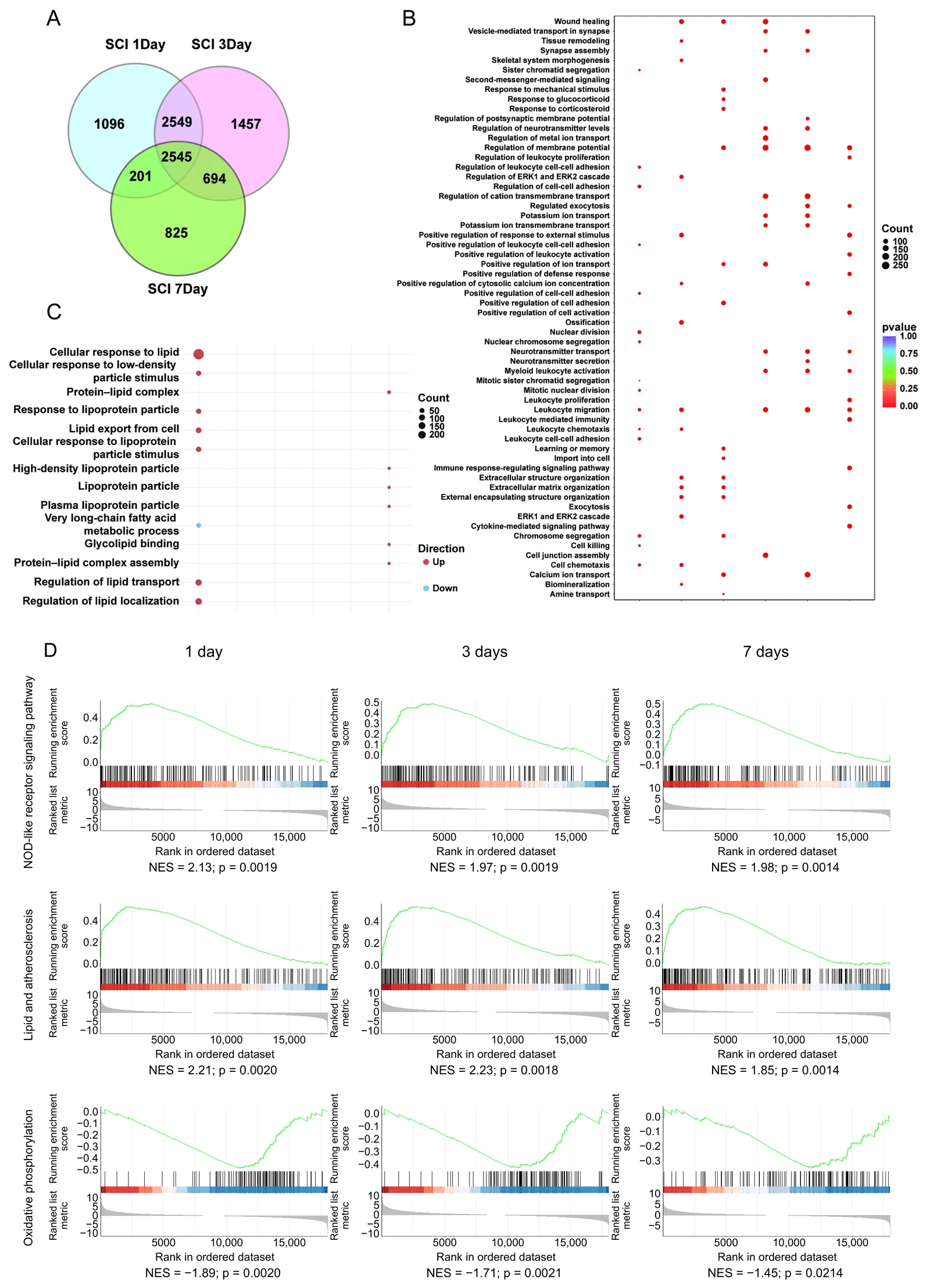
Differentially expressed genes and pathway enrichment analysis after SCI. (**A**). Venn diagram illustrating the overlap of DEGs identified in the 1 d, 3 d, and 7 d groups relative to the control group. Each set includes both upregulated and downregulated DEGs. Different colors correspond to individual time points, and the numbers indicate the gene counts within each region. (**B**). GO enrichment bubble plot showing significantly enriched biological processes across the indicated comparisons. Bubble size represents the number of enriched genes, and bubble color indicates the *p-value* according to the color scale. (**C**). GO enrichment bubble plot focusing on lipid-related biological processes. Red and blue indicate enrichment among upregulated and downregulated genes, respectively, and bubble size represents the number of enriched genes. (**D**). GSEA of selected pathways in the 1 d, 3 d, and 7 d groups compared with the control group.

To further define the molecular basis underlying the candidate lipid alterations described above, we performed temporal transcriptomic profiling of injured spinal cord tissue and complemented this analysis with RT-qPCR validation of key metabolic genes. SCI induced extensive and dynamic transcriptional reprogramming as early as 1 d after injury. DEGs were first identified by separately comparing the 1 d, 3 d, and 7 d post-injury groups with the control group. The three resulting DEG lists were then used for Venn diagram analysis, which revealed a substantial set of DEGs shared across the three post-injury stages, together with time point-specific DEGs (Figure 5A).

To explore the functional significance of this transcriptional remodeling, pathway enrichment analyses were performed. GO analysis consistently revealed significant enrichment of lipid-related biological processes, including lipid biosynthetic process and fatty acid metabolic process, beginning at 1 d after injury and persisting at subsequent time points (Figure 5B). GSEA further demonstrated a sustained transcriptional signature characterized by sustained positive enrichment of innate immune- and lipid metabolism-related gene sets, accompanied by persistent suppression of oxidative phosphorylation (OXPHOS) (Figure 5D). In particular, the NOD-like receptor (NLR) signaling pathway and the lipid and atherosclerosis pathway were significantly enriched. Collectively, these findings indicate that the injured spinal cord rapidly enters a sustained transcriptional state characterized by activation of innate inflammatory signaling and lipid metabolic programs, accompanied by persistent negative enrichment of the OXPHOS gene set.

**Figure 6:**
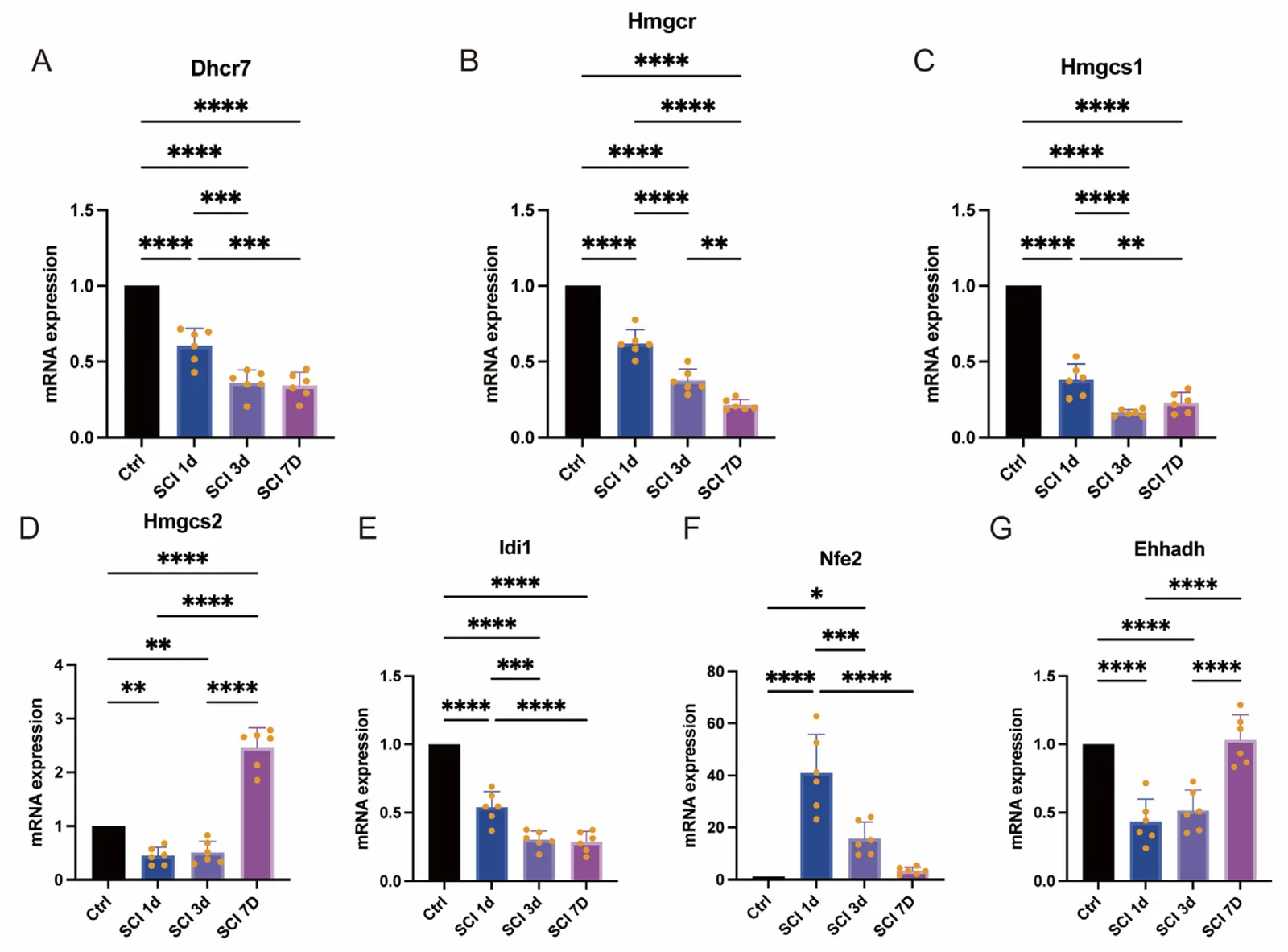
RT-qPCR validation of selected metabolic and injury-responsive genes after SCI. (**A**–**G**). Relative mRNA expression levels of seven selected genes were quantified in spinal cord tissues from the control group and SCI rats at different time points. The panel included six metabolism-related genes (*Dhcr7*, *Hmgcr*, *Hmgcs1*, *Hmgcs2*, *Idi1*, and *Ehhadh*) and the injury-responsive transcription factor *Nfe2*. * *p* < 0.05; ** *p* < 0.01; *** *p* < 0.001; **** *p* < 0.0001. Each orange dot represents a single measurement from an individual rat.

To validate representative transcriptional changes identified by RNA-seq analysis, we examined the expression dynamics of seven selected metabolic and injury-responsive genes in injured spinal cord tissue by RT-qPCR (Figure 6A–G). These analyses showed clear time-dependent changes in the expression of the selected genes after SCI. Genes involved in cholesterol biosynthesis, including *Dhcr7*, *Hmgcr*, *Hmgcs1* and *Idi1*, were rapidly suppressed and remained at low levels through 7 days after SCI. In contrast, pathways related to alternative energy utilization, including mitochondrial ketone body metabolism and fatty acid β-oxidation/peroxisomal metabolism, displayed distinct temporal changes after SCI. *Hmgcs2*, which encodes a mitochondrial enzyme involved in ketone body production, showed reduced expression at 1 and 3 d but increased above the control level at 7 d, suggesting a delayed transcriptional response related to ketone body metabolism. *Ehhadh*, which is involved in fatty acid oxidation and peroxisomal metabolism, showed a biphasic pattern: it was suppressed during the acute phase (1–3 d) and then recovered markedly at 7 d. *Nfe2* also showed a short but strong induction at 1 d, suggesting activation of an early stress-responsive transcriptional program. Together, these validation data indicate stage-dependent changes in metabolic gene expression within the injured spinal cord, characterized by sustained suppression of cholesterol biosynthesis-related genes and delayed changes in genes associated with alternative substrate metabolism after SCI.

## 4. Discussion

In the present study, we applied UPLC-MS/MS-based broad-coverage lipidomics to analyze serum and injured spinal cord tissues from rats with SCI in order to delineate the temporal dynamics of SCI-associated lipidomic alterations and identify candidate serum lipid biomarkers. In parallel, we integrated temporal transcriptomic profiling and RT-qPCR validation of injured spinal cord tissue to define the transcriptional basis of post-injury metabolic remodeling. Our results demonstrated marked alterations in lipid metabolism in both serum and spinal cord tissue after SCI, and identified a panel of potential lipid biomarkers, primarily represented by CEs and LCACs, whose dynamic changes reflected ongoing lipid metabolic reprogramming following injury. Importantly, transcriptomic analyses further revealed sustained activation of innate immune- and lipid metabolism-related pathways together with persistent suppression of OXPHOS. These findings suggest that the observed lipidomic alterations occurred in parallel with local inflammatory activation, metabolic dysregulation, and impaired mitochondrial energy production within the injured spinal cord. Together, these changes point to a close relationship between lipid remodeling and the secondary injury response. SCI can lead to shock, organ dysfunction, and even death, underscoring the importance of defining post-injury abnormalities in cellular and metabolic networks for clinical monitoring and therapeutic intervention [21,22]. Previous SCI biomarker studies have mainly focused on proteins in peripheral serum or cerebrospinal fluid, whereas lipid metabolic remodeling remains less well studied [23]. Cerebrospinal fluid collection is invasive and difficult to use for repeated monitoring in clinical practice. By contrast, serum testing is less invasive and can be repeated over time, which makes it suitable for monitoring neural injury, inflammation, and metabolic changes after SCI [24,25]. In this study, we therefore focused on peripheral serum and integrated these results with lipidomic and transcriptomic data from spinal cord tissue to evaluate temporal associations between the peripheral and tissue-level responses. Metabolic changes within 6–72 h after SCI have been considered important for understanding the early injury response and for assessing injury severity [26,27]. Consistent with this view, we observed marked lipid changes during this early window. Most altered lipids were decreased at 6 h, but the pattern reversed after 1 d. The early serum lipid alterations may also reflect systemic stress and spinal shock-associated autonomic and metabolic responses, including altered hepatic and lipoprotein metabolism, rather than lipid release from the injured spinal cord alone. Lipid changes were predominantly increases at 3 d and 7 d, and the original 14 d volcano plot likewise showed predominant upregulation, with 884 upregulated and 40 downregulated lipids. Thus, the differential lipid profiles indicate early suppression followed by sustained predominant upregulation through 14 d, rather than a late global shift toward downregulation [28,29]. Temporal changes in individual structural and neutral lipid subclasses further indicate remodeling of selected lipid pools [30,31,32]. Temporal transcriptomic analysis showed that SCI triggered large-scale transcriptional reprogramming as early as 1 d after injury; across 1 d, 3 d, and 7 d, a shared core injury-response gene set coexisted with time-dependent transcriptional changes, indicating that metabolic remodeling is initiated during the acute phase and continues to evolve thereafter [33,34].

At the level of lipid remodeling, we observed that structural phospholipids such as PC and PE fluctuated during the early phase after injury and subsequently increased, suggesting rapid reorganization of membrane architecture and signaling lipids [34,35]. Previous studies have reported a significant increase in LPC in the plasma of patients with SCI [16,17,18]. Consistent with these findings, we also detected upregulation of multiple LPC and LPE species beginning at 1 d after SCI in rats. These alterations are consistent with early phospholipase-related membrane lipid remodeling and enhanced inflammatory signaling, suggesting that LPC/LPE-associated metabolism may contribute to disease progression after SCI [36,37]. Notably, TG displayed a temporal pattern distinct from that of membrane phospholipids, with a marked increase at 14 d, suggesting progressive neutral lipid accumulation during the subacute and late phases after injury [30,38]. Transcriptomic profiling further supported this interpretation, as GO analysis consistently showed enrichment of lipid biosynthetic process and fatty acid metabolic process beginning at 1 d after SCI and persisting at later time points, indicating that lipid remodeling was not only evident at the metabolite level but was also sustained at the transcriptional level [34].

One notable finding of this study was that LCACs and CEs did not change synchronously in serum and spinal cord tissue. Instead, alterations in the circulation appeared earlier, whereas accumulation in the injured cord became evident at later time points [32,39]. For LCACs, serum levels increased rapidly during the acute phase after SCI, whereas obvious accumulation in spinal cord tissue was mainly detected from 3 d onward. This temporal difference suggests that serum LCACs may serve as early indicators of SCI-associated metabolic disturbance, although their tissue origin and relationship to spinal cord metabolism remain to be clarified. The transcriptomic results provide complementary molecular context for these observations. GSEA showed that pathways related to lipid metabolism and inflammatory signaling were already enriched at 1 d after injury, while OXPHOS remained suppressed across the examined time window [27,34]. Consistently, *Ehhadh*, which is involved in fatty acid oxidation and peroxisomal metabolism, showed early downregulation followed by partial recovery at later stages. These transcriptional changes are consistent with altered fatty acid oxidation and mitochondrial energy metabolism within the injured spinal cord [40,41]. In this context, incomplete fatty acid oxidation could favor acylcarnitine accumulation, which may explain why circulating LCACs are able to capture acute-phase energetic stress after SCI [42]. In addition, LCAC accumulation has been linked to oxidative stress and inflammatory activation in other neurological disorders. In the context of SCI, the observed LCAC changes may therefore reflect acute metabolic stress, although their possible functional involvement in secondary injury requires direct experimental evaluation [43].

It should be emphasized, however, that elevated acylcarnitine levels are not unique to SCI. Similar changes may also be observed in other conditions associated with stress responses or impaired mitochondrial bioenergetics, such as severe trauma, severe infection, sepsis, ischemia–reperfusion injury, and traumatic brain injury [44,45]. Therefore, a more clinically appropriate strategy would be to regard LCACs as sensitive indicators of SCI-associated disturbances in energy metabolism and to integrate them with neural injury-specific biomarkers, such as GFAP and UCH-L1, or with clinical scoring systems and imaging findings, in order to improve their relative specificity and early discriminatory performance in SCI [23,46].

In addition to LCACs, we further focused on CEs, a lipid class directly linked to lipoprotein transport and cholesterol homeostasis [47,48]. CEs are major components of low-density lipoprotein and key lipid constituents of atherosclerotic plaques, and dysregulation of their circulating levels has been closely associated with multiple metabolic disorders, including coronary artery disease, non-alcoholic fatty liver disease, and chronic kidney disease. In the present study, multiple CEs in injured spinal cord tissue showed an overall progressive accumulation, whereas serum CEs from the same animals exhibited a biphasic pattern characterized by an initial transient decrease followed by a gradual rebound. We speculate that this divergence reflects distinct dominant drivers in the lesion site and peripheral circulation. In the acute lesion environment, large amounts of myelin and membrane lipid debris are released and engulfed by microglia and macrophages, which may esterify free cholesterol into CEs for intracellular storage, thereby contributing to sustained tissue accumulation. In parallel, acute stress and systemic inflammation may transiently reduce circulating CEs by remodeling lipoprotein metabolism or altering hepatic synthesis and export. As systemic lipid homeostasis is gradually re-established, serum CE levels rebound. This serum-tissue dissociation is also concordant with the sustained enrichment of the lipid and atherosclerosis pathway in the transcriptomic data, suggesting that SCI is associated not only with local dysregulation of cholesterol handling but also with a broader lipid-inflammatory coupling state [30,49]. Notably, tissue CE accumulation does not necessarily indicate enhanced de novo cholesterol biosynthesis. In the context of suppressed cholesterol biosynthetic genes within the injured spinal cord, CE accumulation may instead reflect esterified storage of free cholesterol derived from myelin and membrane debris after uptake by phagocytic cells, or insufficient local cholesterol efflux [50,51].

The integrated transcriptomic and RT-qPCR results provided complementary molecular context for the lipidomic changes observed in this study. GO analysis and GSEA indicated that, after SCI, the injured spinal cord rapidly entered a sustained transcriptional state involving innate inflammatory activation, lipid metabolic remodeling, and persistent suppression of OXPHOS [34]. Consistent with these pathway-level findings, RT-qPCR validation showed marked suppression of several cholesterol biosynthesis-related genes, including *Dhcr7, Hmgcr, Hmgcs1* and *Idi1* from the early phase to 7 d after injury [50]. By contrast, *Hmgcs2* expression was reduced at 1 and 3 d but increased above the control level at 7 d, suggesting a delayed transcriptional response related to ketone body metabolism. Similarly, the recovery of *Ehhadh* expression at 7 d indicated a late change in fatty acid oxidation- and peroxisomal metabolism-related gene expression [41,52]. Overall, these results indicate stage-dependent changes in metabolic gene expression within the injured spinal cord, characterized by sustained suppression of cholesterol biosynthesis-related genes and delayed alterations in genes associated with ketone body metabolism and fatty acid oxidation. From a broader perspective, the concurrent changes in lipid metabolic pathways and inflammatory programs suggest that lipid metabolic dysregulation may form an important molecular background for secondary injury progression after SCI. However, whether this relationship is causal remains to be determined by further functional studies [32].

Several limitations should be considered when interpreting these findings. First, the study was performed in a rodent SCI model. Although the experimental conditions were carefully controlled, species-related differences cannot be ignored, and validation in large human cohorts will be required [53]. Second, there is still no universally standardized workflow for mass spectrometry-based detection of serum lipid subclasses, which may limit direct comparison across platforms and laboratories. Third, we did not directly investigate the tissue sources of the circulating lipid alterations observed in this study. Serum LCAC levels may be influenced by metabolic activity in the liver, skeletal muscle, adipose tissue, and immune cells, whereas circulating CE levels may also be affected by hepatic and lipoprotein metabolism [54,55]. In addition, we did not assess blood–spinal cord barrier (BSCB) permeability or its temporal course after SCI. Therefore, the extent to which BSCB disruption contributes to circulating lipid changes or mediates exchange between the injured spinal cord and circulation remains uncertain. Future studies incorporating isotope tracing, organ-specific metabolic flux analysis, and lipidomic profiling of other peripheral tissues will be needed to clarify the tissue sources of these circulating lipid alterations and their relationship to metabolic changes in the injured spinal cord. Fourth, although the transcriptomic and RT-qPCR data provided mechanistic clues linking lipidomic alterations with inflammatory activation and impaired energy metabolism, we did not directly manipulate LCAC or CE levels or examine whether changes in these lipids affect mitochondrial function, tissue damage, inflammation, or neurological recovery after SCI. Consequently, LCACs and CEs are best regarded at this stage as candidate biomarkers associated with SCI-related metabolic remodeling. Targeted genetic, pharmacological, or metabolic intervention studies will be needed to determine whether these lipids have functional roles in secondary SCI pathology. From a translational perspective, one strength of this study is that three complementary datasets were generated in parallel, including serum lipidomics, spinal cord tissue lipidomics, and spinal cord transcriptomics. Serum profiling is suitable for minimally invasive and repeated monitoring, whereas tissue lipidomics helps characterize the local metabolic burden within the injured cord. In addition, transcriptomic analysis and RT-qPCR validation provide complementary molecular context for the metabolic alterations observed at the lipid level. Using this multi-omics strategy, we identified LCACs and CEs as candidate serum lipid signatures with distinct temporal profiles across post-injury stages. Parallel lipidomic and transcriptomic changes revealed coordinated temporal remodeling in serum and injured spinal cord tissue. These findings support further evaluation of LCACs and CEs as minimally invasive markers of SCI-associated metabolic changes; however, their tissue origins, relationship to local spinal cord pathology, and potential modulation by BSCB permeability remain to be determined.

## Figures and Tables

**Table 1:** Primer sequences used for RT-qPCR.

Gene	Forward Primer (5′→3′)	Reverse Primer (5′→3′)
*Dhcr7*	AGGCTGGATCTCAAGGACAAT	GCCAGACTAGCATGGCCTG
*Hmgcr*	AGCTTGCCCGAATTGTATGTG	TCTGTTGTGAACCATGTGACTTC
*Hmgcs1*	AACTGGTGCAGAAATCTCTAGC	GGTTGAATAGCTCAGAACTAGCC
*Hmgcs2*	GAAGAGAGCGATGCAGGAAAC	GTCCACATATTGGGCTGGAAA
*Idi1*	ACCAGCCATCTTGATGAAAAACA	CAGCAACTATTGGTGAAACAACC
*Nfe2*	TCCTCAGCAGAACAGGAACAG	GGCTCAAAAGATGTCTCACTTGG
*Ehhadh*	ATGGCTGAGTATCTGAGGCTG	GGTCCAAACTAGCTTTCTGGAG

## Data Availability

The data presented in this study are available from the corresponding author upon reasonable request.
